# Snare-tip soft coagulation to treat esophageal stent epithelial hyperplasia

**DOI:** 10.1055/a-2050-7431

**Published:** 2023-03-23

**Authors:** Sunil Gupta, Anthony Whitfield, Andrew Tang, Eric Y. T. Lee, Stephen J. Williams, Nicholas G. Burgess, Michael J. Bourke

**Affiliations:** 1 Westmead Hospital, Department of Gastroenterology and Hepatology, Sydney, Australia; 2 University of Sydney, Westmead Clinical School, Sydney, Australia

A 65-year-old man with a spontaneous esophageal perforation (Boerhaave syndrome) was managed with a partially covered metal stent (23 mm wide × 12.5 mm long). After an indwelling time of 8 weeks, endoscopic removal was attempted. Significant epithelial hyperplasia was noted at the proximal and distal ends of the stent ( Fig. 1 a, b ). Despite treatment with argon plasma coagulation (APC) combined with stent-in-stent insertion 1 , attempts at removal failed.

**Fig. 1:**
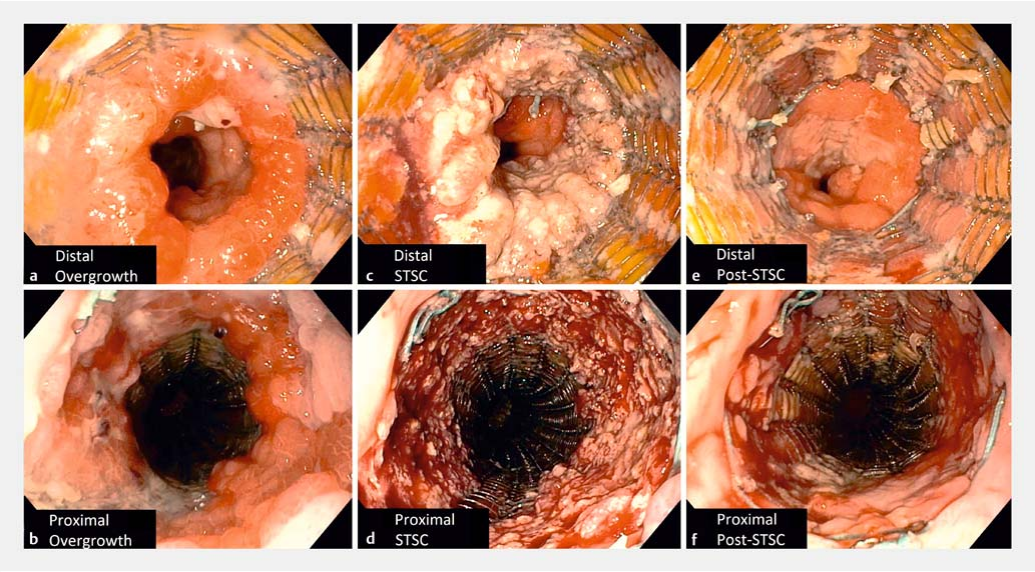
Application of snare-tip soft coagulation (STSC) to epithelial hyperplasia. **a, b** Proximal and distal ends of the partially covered metal stent demonstrating epithelial hyperplasia. **c, d** Ablated epithelial hyperplasia following STSC. **e, f** Post-STSC and stent-in-stent therapy revealing near complete resolution of epithelial hyperplasia.

We proceeded to utilize a dedicated 10-mm hot snare to perform snare-tip soft coagulation (STSC; Effect 4, 80 Watts; ERBE VIO300 D) (Erbe Elektromedizin, Tübingen, Germany) to ablate the epithelial hyperplasia ( Video 1 ). A uniform field of ablated tissue was obtained ( Fig. 1 c, d ). To promote sloughing of the ablated tissue, a fully covered metal stent was placed within the pre-existing stent. After 2 weeks, the inner stent was removed, revealing near-complete clearance of the epithelial hyperplasia ( Fig. 1 e, f ). After a small amount of additional STSC at the proximal margin, stent removal was easily accomplished with rat-toothed forceps.

**Video 1** Snare-tip soft coagulation to treat esophageal stent epithelial hyperplasia.


While STSC was developed as a measure to reduce recurrent adenoma post colorectal endoscopic mucosal resection 2 , its favorable properties render STSC invaluable in other settings. For example, when compared to other modalities such as APC, direct contact with the snare tip enables uniform delivery of energy to complete destruction of the target tissue 3 . Furthermore, a peak voltage of 190 V results in desiccation. The ensuing lack of carbonization and charring facilitates reliable energy delivery. Lastly, because resistance to current flow increases exponentially as tissue is desiccated, excessive current flow is limited. Subsequent termination of energy transfer has the potential to prevent deep tissue injury 4 . While placement of the inner stent may have contributed to tissue destruction, we believe that STSC is a safe, cost-effective, and efficacious way to treat esophageal stent epithelial hyperplasia.

Endoscopy_UCTN_Code_TTT_1AO_2AZ

## Notes

Endoscopy E-Videos

https://eref.thieme.de/e-videos
*Endoscopy E-Videos* is an open access online section, reporting on interesting cases and new techniques in gastroenterological endoscopy. All papers include a high quality video and all contributions are freely accessible online. Processing charges apply, discounts and wavers acc. to HINARI are available. This section has its own submission website at https://mc.manuscriptcentral.com/e-videos
